# Diverse Profile of Fermentation Byproducts From Thin Stillage

**DOI:** 10.3389/fbioe.2021.695306

**Published:** 2021-07-15

**Authors:** Nathaniel W. Fortney, Nathaniel J. Hanson, Paula R. F. Rosa, Timothy J. Donohue, Daniel R. Noguera

**Affiliations:** ^1^Great Lakes Bioenergy Research Center, University of Wisconsin-Madison, Madison, WI, United States; ^2^Wisconsin Youth Apprenticeship Program, Department of Workforce Development, Madison, WI, United States; ^3^Department of Chemical Engineering, Federal University of São Carlos, São Carlos, Brazil; ^4^Department of Bacteriology, University of Wisconsin-Madison, Madison, WI, United States; ^5^Department of Civil and Environmental Engineering, University of Wisconsin-Madison, Madison, WI, United States

**Keywords:** fermentation, microbial communities, valorization, chain elongation, thin stillage

## Abstract

The economy of biorefineries is influenced not only by biofuel production from carbohydrates but also by the production of valuable compounds from largely underutilized industrial residues. Currently, the demand for many chemicals that could be made in a biorefinery, such as succinic acid (SA), medium-chain fatty acids (MCFAs), and lactic acid (LA), is fulfilled using petroleum, palm oil, or pure carbohydrates as raw materials, respectively. Thin stillage (TS), the residual liquid material following distillation of ethanol, is an underutilized coproduct from the starch biofuel industry. This carbon-rich material has the potential for chemical upgrading by microorganisms. Here, we explored the formation of different fermentation products by microbial communities grown on TS using different bioreactor conditions. At the baseline operational condition (6-day retention time, pH 5.5, 35°C), we observed a mixture of MCFAs as the principal fermentation products. Operation of a bioreactor with a 1-day retention time induced an increase in SA production, and a temperature increase to 55°C resulted in the accumulation of lactic and propionic acids. In addition, a reactor operated with a 1-day retention time at 55°C conditions resulted in LA accumulation as the main fermentation product. The prominent members of the microbial community in each reactor were assessed by 16S rRNA gene amplicon sequencing and phylogenetic analysis. Under all operating conditions, members of the *Lactobacillaceae* family within *Firmicutes* and the *Acetobacteraceae* family within Proteobacteria were ubiquitous. Members of the *Prevotellaceae* family within *Bacteroidetes* and *Lachnospiraceae* family within the *Clostridiales* order of *Firmicutes* were mostly abundant at 35°C and not abundant in the microbial communities of the TS reactors incubated at 55°C. The ability to adjust bioreactor operating conditions to select for microbial communities with different fermentation product profiles offers new strategies to explore and compare potentially valuable fermentation products from TS and allows industries the flexibility to adapt and switch chemical production based on market prices and demands.

## Introduction

Conventional production of bioethanol utilizing starch from cereal grains (e.g., corn, wheat, and barley) is a mature industry, with over 14 billion gallons of ethanol produced annually in the United States (United States Department of Energy, 2016; United States Department of Agriculture, 2020). This currently comprises almost 75% of the United States biofuel production market (Moriarty et al., 2020) and is projected to be a significant contribution to bioenergy in the near term. In a typical starch bioethanol plant, grain is milled and chemically and enzymatically pretreated, and the resulting carbohydrate monomers are fermented to ethanol by yeast (Reis et al., 2017; Gronchi et al., 2019). The residue from bioethanol production is called whole stillage, which is typically separated into thin stillage (TS) and wet distillers’ grains (WDGs). TS is the residual liquid material, typically containing 5–10% solids, following fermentation, ethanol distillation, and centrifugation of whole stillage. WDG is the solid residue after centrifugation (Reis et al., 2017). We are interested in evaluating microbial routes to valorize TS by fermentation into valuable products.

Currently, bioethanol facilities recycle about 15% of the TS as backset process water for the next batch of starch bioethanol fermentation (McAloon et al., 2000; Kim et al., 2008). The remaining TS is typically condensed through a series of evaporators and dried along with WDG to produce dried distillers’ grains with solubles (DDGS). Together, these are the major coproducts to bioethanol production and represent a large fraction of unconverted biomass from the initial crop feedstock (Andersen et al., 2015). TS, WDG, and DDGS are marketed as high-protein animal feed (Mustafa et al., 1999; Birkelo et al., 2004; Gillespie et al., 2013; Lupitskyy et al., 2015; Sekhon et al., 2018) and are important to the overall economics of a bioethanol plant (Reis et al., 2017; Moriarty et al., 2020). Alternatively, anaerobic digestion can be used to produce biogas, which is used to offset the gas and electricity requirements of the ethanol plant (Wilkie et al., 2000; United States Department of Energy, 2016). Volumetrically, TS is produced at a rate of 15–20 L per L ethanol (Loehr and Sengupta, 1985; Wilkie et al., 2000; Reis et al., 2017) in a typical 50 million gallon (189,000 m^3^) per year starch ethanol plant; and accounting for recycled backset, this still results in 2–3 million m^3^ of TS produced annually. Thus, there is a large supply of TS for valorization into high value products, with concomitant economic benefits and waste reduction for bioethanol plants (Chatzifragkou et al., 2015).

Producing high-value chemicals from agro-industrial residues, such as TS, has the potential to improve the overall economics of bioenergy production and contribute toward a greener global energy and chemical market. The composition of TS is dependent on the characteristics of the incoming grain, the efficiency of starch hydrolysis during pretreatment, the fermentation efficiency, and any metabolic products of the ethanologenic fermentation. Corn starch hydrolysis, for example, is an incomplete process; and as much as half of the incoming chemical energy ends up in TS as unfermented carbohydrates, typically oligomers of two or more glucose subunits (Kim et al., 2008; Andersen et al., 2015; Bilskey et al., 2020). Unfermented sugars, yeast byproducts (e.g., glycerol), and organic acids result in a chemically and energetically rich material (Wilkie et al., 2000; Lee et al., 2011; Reis et al., 2017). Several fermentation products have been identified as targets for production from TS and other agro-industrial residues (Werpy et al., 2004; Agler et al., 2011; Kleerebezem et al., 2015; Biddy et al., 2016). For instance, medium-chain fatty acids (MCFAs) are used in consumer products and also have the potential to serve as precursors for drop-in biofuels (Agler et al., 2011). Succinic acid (SA) is a useful platform chemical for the synthesis of other high-value commodity chemicals. Historically, the majority of SA production came from petroleum (Rogers et al., 2013; Biddy et al., 2016), but demand for and production of SA from renewable sources are increasing (Werpy et al., 2004; Carlson et al., 2016; Nghiem et al., 2017; Kuenz et al., 2020). The industrial demand for lactic acid (LA) is large (Harmsen et al., 2014) and satisfied mostly from commodity sugars and starch (Ghaffar et al., 2014; Biddy et al., 2016; Zhang et al., 2017). A growing demand for the LA-based polymer polylactic acid (PLA) has resulted in a renewed interest in developing strategies to selectively produce each of the LA stereoisomers independently (Pang et al., 2010; Biddy et al., 2016; Zhang et al., 2017).

The use of TS to contribute to some of these and other product markets, in addition to animal feed, is well recognized (Reis et al., 2017). Some studies have focused on recovering more ethanol from TS by engineering bacteria to utilize the glycerol produced by ethanologenic yeast during primary fermentation (Gonzalez et al., 2010). In addition, the use of TS as a feedstock for caproic acid production has been recently studied (Andersen et al., 2014, 2015, 2017; Carvajal-Arroyo et al., 2019).

Here, we used TS from a corn starch bioethanol plant as a feedstock to test the hypotheses that (1) TS can be biologically upgraded to different higher-value fermentation products and (2) altering the operating conditions of a bioreactor will alter the major fermentation products. Five different experimental conditions were studied to determine the effect of reactor conditions on TS fermentation. Operating under mesophilic conditions (35°C), the microbial community converted TS to primarily short-chain fatty acids (SCFAs) and MCFAs. Decreasing the solids retention time (SRT) induced a change in product profile resulting in greater SA production. A change in bioreactor in temperature to thermophilic conditions (55°C) resulted in accumulation of propionic acid and LA, while thermophilic reactor conditions with short SRT resulted in primarily LA accumulation. By showing the differences in fermentation product accumulation when a single source of inoculum and feedstock is used, this study provides an important comparison of the range of coproducts that can be produced from TS by only altering the bioreactor conditions.

## Materials and Methods

### Source of Thin Stillage

ICM, Inc., provided approximately 125 L of frozen TS, which was received in August 2019 from its affiliated ethanol plant, ICM Biofuels, LLC, St. Joseph, MO, United States. The ethanol process is detailed elsewhere (Dieker et al., 2016). In this plant, corn is milled, water and enzymes are added to the milled corn, and corn is fermented to ethanol with a strain of *Saccharomyces cerevisiae*. TS is obtained following ethanol distillation and centrifugation of the whole stillage to remove most residual corn solids. TS was thawed at room temperature, split into aliquots of ca. 20 L, and frozen again at −20°C until needed.

Solids-removed TS (SR-TS) was prepared by thawing an aliquot of TS at room temperature for 1 day, allowing solids to settle for at least 2 days at 4°C and pumping the supernatant off into a new container. By volume, approximately 2/3 of the original TS was the less dense material that constituted the SR-TS, leaving about 1/3 higher density material, which settled out and was left as residual solids not used in this study. Measured properties of TS and SR-TS are presented in Table 1.

**TABLE 1:** Composition of influent thin stillage.

	**Thin stillage (TS)^i^**	**Solids-removed thin stillage (SR-TS)^i^**
pH	4.56	4.44
Total ammonium–N^a^ (g L^–1^)	0.11 ± 0.04	0.28 ± 0.2
Phosphate-P (g L^–1^)	1.0 ± 0.1	1.0 ± 0.2
TSS^b^ (g L^–1^)	34.0 ± 1.3	5.5 ± 0.2
VSS^c^ (g L^–1^)	33.1 ± 1.3	4.3 ± 2.0
COD^d^_Total_ (g COD L^–1^)	130 ± 20	67 ± 6
COD_Soluble_ (g COD L^–1^)	67 ± 8	66 ± 9
Total soluble carbohydrates^e^ (g COD L^–1^)	4.7 ± 0.6	4.4 ± 0.6
Glycerol (g COD L^–1^)	23 ± 4	24 ± 3
Lactic acid (g COD L^–1^)	4.4 ± 1.1	4.2 ± 0.6
Other carboxylates^f^ (g COD L^–1^)	2.3 ± 0.4	2.1 ± 0.7
Soluble protein (g COD L^–1^)	2.1 ± 0.4	1.8 ± 0.5
Insoluble protein^g^ (g COD L^–1^)	0.7 ± 0.4	0.7 ± 0.4
Other soluble^h^ (g COD L^–1^)	30 ± 13	31 ± 5

*^a^Measured total ammonium nitrogen (sum of NH_4_^+^–N and NH_3_–N) in the feedstock indicated a potential nutrient deficiency for fermentation. This was solved by the addition of NH_4_OH as the base for pH control.*

*^b^Total suspended solids.*

*^c^Volatile suspended solids.*

*^d^Chemical oxygen demand.*

*^e^Soluble carbohydrate monomers and oligomers.*

*^f^Other measured carboxylic acids include pyruvic acid, succinic acid, short-chain fatty acid (SCFA), and medium-chain fatty acid (MCFA).*

*^g^Determined by the difference between total protein and soluble protein.*

*^h^Quantifiable by chemical oxygen demand (COD) not associated with other analytical measurements.*

*^i^Reported results are average and standard deviation of six replicates.*

### Mixed Microbial Culture Fermentation Experiments

An initial bioreactor was inoculated with sludge collected from the acid-phase digester of a two-phase anaerobic digestion process at the Madison Metropolitan Sewerage District’s Nine Springs Facility in Madison, WI, United States. The bioreactor consisted of a 3-L reactor vessel and an ez-Control unit (Applikon Biotechnology, Inc., Dover, NJ, United States). At start-up, the reactor was filled with 750 ml of inoculum and 750 ml of TS. The reactor was mixed at 150 rpm using a direct-drive motor, temperature was maintained at 35°C, and pH was controlled at 5.5 using 10% NH_4_OH and 1 M of H_3_PO_4_. The use of NH_4_OH for pH control was done to guarantee that the culture was not nitrogen deficient. Following reactor fill, volume was reduced and maintained at 1 L by two peristaltic pumps (Watson-Marlow, Ltd., Wilmington, MA, United States) controlling influent and effluent and operating semi-continuously (feeding every 20 min), pumping a total of 167 ml per day into and out of the reactor. These pumping rates maintained a 6-day SRT, which was equal to the hydraulic retention time (HRT). Other bioreactors in this study originated from this initial bioreactor, as described below.

### Variation in Experimental Bioreactor Fermentation Conditions

A total of five experimental bioreactor conditions were tested to explore the effects of influent characteristics, SRT, temperature, and pH on the portfolio of fermentation products. Experimental conditions are detailed in Table 2. In the first experimental condition, the reactor was fed TS for about 90 days (experiment is hereafter referred to as R1_TS_). In the second experimental condition (R2_SR–TS_), the same reactor vessel and operational conditions were maintained, but the influent was changed from TS to SR-TS to evaluate whether removing solids would influence the resulting fermentation products.

**TABLE 2:** Bioreactor operating conditions.

	**Experiment**				
**Parameter**	**R1_TS_**	**R2_SR–TS_**	**R3_LowSRT_**	**R4_T–pH_**	**R5_T–pH–LowSRT_**
Inoculum source	Acid-phase anaerobic digester sludge	R1_TS_	R2_SR–TS_	R2_SR–TS_	R4_T–pH_
Influent	TS	SR-TS	SR-TS	SR-TS	SR-TS
Volume (ml)	1,000	1,000	150	260	150
SRT (days)	6	6	1	6	1
Temperature (°C)	35	35	35	55	55
pH	5.5	5.5	5.5	5.0	5.0

*SRT, solids retention time.*

All additional bioreactor experiments were fed SR-TS and operated with smaller volumes (in 400-ml glass vessels) using a Multifors 2 parallel bench-top bioreactor system (Infors USA, Inc., Annapolis Junction, MD, United States). The third experimental condition tested was reduction of the SRT (R3_LowSRT_), in which an aliquot of the R2_SR–TS_ was transferred to a new vessel and the SRT was gradually reduced from 6 days (25 ml exchanged daily out of 150-ml working volume) to 1 day (150 ml exchanged daily) by increasing pumping duration to accommodate stepwise 1-day SRT reductions, each time holding pumping constant for approximately 10 days and then implementing another SRT reduction step.

In a fourth experiment, we tested thermophilic operation with reduced pH (R4_T–pH_), while maintaining a 6-day SRT. In this experiment, the temperature was increased to 55°C, and the pH decreased to 5.0. Thermophilic operation with a reduced SRT was tested in the fifth experiment (R5_T–pH–LowSRT_), in which starting with the operational conditions of R4_T–pH_, the pumping rates were adjusted to reduce the SRT to 1 day over a 20-day operational period.

### Sample Collection and Analysis

Samples were collected from all bioreactors periodically for biomass and chemical analyses. Biomass was collected by centrifuging four 1.5-ml aliquots of bioreactor contents at 10,000*g* for 10 min. Supernatant was removed, and pellets were stored at −80°C for subsequent DNA extraction. Total and soluble [i.e., filtered through a 0.2 μm Whatman^®^ Puradisc 25 polyethersulfone (PES) membrane syringe filter (GE Healthcare, Chicago, IL, United States)] chemical oxygen demand (COD) assays were conducted using Hach High-Range COD2 Hg-Free Digestion Vials (Hach, Loveland, CO, United States) following the manufacturer’s protocol (Method 8000, Hach). The difference between the total and soluble COD (i.e., the insoluble COD) was used as an indicator of biomass in samples from bioreactors fed SR-TS. Total and soluble protein was measured using the Pierce^TM^ BCA Assay Kit and Compat-Able^TM^ Protein Assay Preparation Reagent Set (Thermo Fisher Scientific, Waltham, MA, United States) following the manufacturer’s protocol for a 96-well microtiter plate. Total ammonium nitrogen (TAN; the sum of NH_4_^+^–N and NH_3_–N) was measured using the salicylate method (Method 10023, Hach) and Ammonia Salicylate and Cyanurate Reagent Powder Pillows (Hach). Free ammonia nitrogen (FAN) was estimated using formulae presented in Capson-Tojo et al. (2020). The protocol was modified for testing in a 96-well microtiter plate. Microtiter plates were read using a Tecan Infinite M1000 plate reader and Magellan software (Tecan US, Inc., Morrisville, NC, United States). Phosphate concentration was analyzed using the ascorbic acid method (Method 8048, Hach) and PhosVer 3^®^ Phosphate Reagent Powder Pillows (Hach). With the exception of samples collected for biomass and total COD, all samples were filtered using a 0.2-μm syringe filter (Whatman^®^ Puradisc 25 PES membrane, GE Healthcare) prior to analysis.

The concentration of carbohydrates (glucose, xylose, and cellobiose), organic acids [formic, acetic (C2), LA, pyruvic acid, and SA], and sugar alcohols (glycerol and xylitol) in the bioreactor effluents were determined by high-performance liquid chromatography (HPLC) with an Agilent 1260 Infinity HPLC system and refractive index detector (Agilent Technologies, Inc., Palo Alto, CA, United States). Analytes were separated using a Bio-Rad 300 × 7.8 mm Aminex HPX-87H column and Cation-H guard column (Bio-Rad, Inc., Hercules, CA, United States) at 50°C with 0.02 N of H_2_SO_4_ mobile phase and 0.5 ml min^–1^ flow rate. Total soluble carbohydrates in the influent and in a subset of bioreactor samples were quantified using acid hydrolysis and alditol acetate derivatization (Foster et al., 2010). The free monomeric carbohydrates resulting from the hydrolysis of oligomeric carbohydrates were quantified using the Megazyme D-fructose/D-glucose kit (Megazyme, Wicklow, Ireland) rather than by HPLC due to coeluting compounds in the HPLC method used for quantification of glucose, xylose, and cellobiose described above. The oligomeric carbohydrate concentration was determined by the difference of total soluble and monomeric carbohydrates. Headspace solid-phase microextraction was used to collect the SCFAs propionic (C3), butyric (C4), and valeric (C5) acids; the MCFAs caproic (C6), enanthic (C7), and caprylic (C8) acids; and ethanol from liquid samples and were analyzed by gas chromatography–mass spectrometry (GC-MS) using an Agilent 7890A GC system (Agilent Technologies, Inc.) equipped with an L-PAL3 heated, agitating autosampler with SPME headspace syringe (LECO Corporation, St. Joseph, MI, United States), and a Pegasus BT TOF-MS detector (LECO Corp.). ChromaTOF^®^ v5.40.12 (Leco Corp.) software was used for MS data acquisition and analysis. The concentration of hydrogen gas (H_2_) and methane (CH_4_) in the bioreactor headspace was analyzed using a Shimadzu GC-2014 (Shimadzu Scientific Instruments, Inc., Columbia, MD, United States).

### DNA Extraction, Sequencing, and Analysis

DNA was extracted from biomass pellets using either a phenol-chloroform extraction method (Scarborough et al., 2018a), or the QIAGEN DNeasy^®^ PowerSoil^®^ Pro kit (QIAGEN, Inc., Germantown, MD, United States), according to manufacturer’s instructions. DNA was submitted to the University of Wisconsin Biotechnology Center (UWBC^1^) for paired-end, 2 × 300 bp Illumina MiSeq (Illumina, Inc., San Diego, CA, United States) 16S rRNA gene amplicon sequencing using primers targeting the V3–V4 region of the 16S rRNA gene (Klindworth et al., 2013).

Raw 16S rRNA gene amplicon sequences were processed through QIIME (v1.9.1; Caporaso et al., 2010). Briefly, forward and reverse reads were paired, quality trimmed using a Phred score cut-off of 20, and split by sample barcode ID. Chimeric sequences were detected using the *usearch61* method (Edgar, 2010). Filtered sequences from all samples were concatenated into a single input file; open reference operational taxonomic units (OTUs) were picked using the *uclust* method (Edgar, 2010). Taxonomic information was assigned to OTUs by aligning representative sequences to the SILVA database (release 138.1^2^) (Quast et al., 2012) using a cut-off of 99%. OTU IDs are assigned as GenBank accession numbers of reference sequences from the SILVA database, or generated *de novo* in the cases where representative sequences failed to align to the database.

Operational taxonomic units were normalized to the fewest number of reads per sample (*n* = 30,354 reads). Beta diversity was calculated using unweighted UniFrac metrics (Lozupone and Knight, 2005; Lozupone et al., 2011). The pairwise distinction between the stable microbial community from each reactor condition was tested using the analysis of similarities (ANOSIM; Clarke, 1993). The ANOSIM test statistic *R* is on a scale of 0–1, where values closer to 0 indicate that the two sample groups are indistinguishable, and values closer to 1 indicate greater distinction between groups.

Relative abundance of OTUs was visualized in R (v3.6.0), using the Superheat (v1.0.0) package (Barter and Yu, 2018). Abundant OTU DNA sequences were aligned using MUSCLE (v3.8.31) (Edgar, 2004), and phylogenetic trees were generated in RAxML (v8.2.11) (Stamatakis, 2014) using the GTRGAMMA method and visualized using FigTree (v1.4.4^3^).

## Results

### Fermentation Experiments

Glycerol, carbohydrates, and LA were the most abundant soluble substrates identified in TS and SR-TS (Table 1). Together, they represented 48% of the soluble COD, with glycerol being the most abundant substrate and accounting for ca. 35% of the soluble COD (Table 1). The soluble protein concentration in TS and SR-TS was low, ca. 3% of the soluble COD (Table 1). The reactors fed with TS and SR-TS were operated for different periods of time, ranging from 66 to 250 days (Supplementary Figure 1). In all cases, a period of reactor stability, defined by a relatively stable concentration of products measured in the bioreactor effluents and lasting about 50 days, was identified and used to compare the results obtained from reactors operated under different conditions.

**FIGURE 1:**
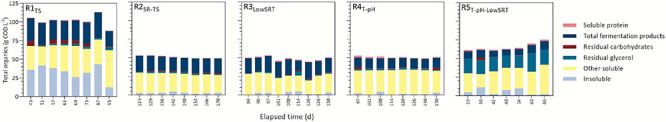
Daily concentration of influent feed converted to fermentation products during indicated periods of reactor stability in reactors ran under different operating conditions. Insoluble portion of the total organics represents the difference between total and soluble COD in the effluent and can be inferred to be accumulated microbial biomass, with the exception of R1_TS_, which also contains influent-derived solids. The other soluble portion represents the measured soluble COD that was not accounted for with other analytical methods plus metabolites present at trace concentrations. Residual carbohydrates are the sum of unconsumed glucose, xylose, and cellobiose in the effluent. Total fermentation products represent the sum of all carboxylic acids and ethanol.

**FIGURE 2:**
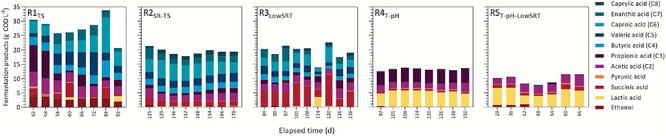
Concentration of observed fermentation products during indicated periods of reactor stability.

During the period of reactor stability, all reactors degraded influent substrates and produced fermentation products (Figure 1). Efficient consumption of glycerol in all bioreactors was consistent with previous fermentation studies (Carlson et al., 2016; Chen et al., 2016; Murakami et al., 2016; Gonzalez-Garcia et al., 2017; Hastati et al., 2017; Veras et al., 2019; Kuenz et al., 2020; Zheng et al., 2021). One exception to this trend is in R5_T–pH–LowSRT_, where only approximately a quarter of the glycerol was consumed. LA, a known substrate for chain elongation (Prabhu et al., 2012; Louis and Flint, 2016; Candry et al., 2020a; Scarborough et al., 2020), was readily consumed in the MCFA-producing bioreactors (R1_TS_, R2_SR–TS_, and R3_LowSRT_, Figure 2). Protein degradation was also evident in some bioreactors (R1_TS_ and R2_SR–TS_, Figure 1) where less than half the influent concentration of protein remained in the effluent (0.5–0.8 g COD L^–1^), while in other bioreactors (R3_LowSRT_, R4_T–pH_, and R5_T–pH–LowSRT_), effluent soluble protein concentrations were 1.2–1.5 g COD L^–1^ compared with the influent concentration of 1.8 g COD L^–1^. The fraction of effluent COD that corresponded to identified and measured fermentation products during the periods of stable operation ranged from as low as 13% in R5_T–pH–LowSRT_ to as high as 50% in R3_LowSRT_ (Figure 1). In addition to residual substrate and fermentation products, a fraction of effluent COD was insoluble (Figure 1). This fraction was greater in R1_TS_ than in the other bioreactors due to this reactor being the only one fed TS instead of SR-TS. However, while the insoluble COD represented 49% of the total COD in TS, it was only about 30% in the effluent of R1_TS_, indicating that a fraction of the solids in TS was hydrolyzed in this bioreactor. The contribution of the hydrolyzed solids to fermentation products in R1_TS_ is reflected in the larger amount of fermentation products in this bioreactor compared with the other bioreactors (Figure 2). The insoluble COD in the bioreactors fed SR-TS accounted for approximately 10% of the total effluent COD (Figure 1), and since SR-TS had most solids removed, this represents the amount of microbial biomass accumulating in the bioreactors.

A comparison of fermentation products measured during the periods of stable operation is presented in Figure 2. Although each bioreactor experiment produced a mixture of different fermentation products, a primary product or class of products was observed under each different set of operating conditions. The total amount (ca. 28 g COD L^–1^) of fermentation products was the greatest in R1_TS_ due to the greater total COD in the influent of this reactor. The R1_TS_ reactor exhibited the largest number of fermentation products. During the stable period of operation (Figure 2), a range of fatty acids from C2 to C7 were detected in the effluent of R1_TS_, in addition to SA, LA, pyruvic acid, and ethanol. The most abundant types of fermentation products in the R1_TS_ reactor were SCFA (C2–C5) and MCFA (C6–C8). The SCFA accounted for 14.7 ± 3.7 g COD L^–1^ (14.4 ± 3.5% of effluent COD; *n* = 8), and the MCFA added up to 7.6 ± 3.4 g COD L^–1^ (7.4 ± 2.9% of effluent COD) in the R1_TS_ reactor. SA and ethanol were also abundant in the R1_TS_ reactor, accounting for 1.9 ± 1.7 g COD L^–1^ (1.8 ± 1.5%) and 3.1 ± 0.6 g COD L^–1^ (3.0 ± 0.5%) of the effluent COD, respectively. Gas production was observed in R1_TS_, where a 1-L gas sample bag (FlexFoil PLUS, SKC, Inc., Eighty Four, PA, United States) inflated about once every week. To evaluate whether H_2_ was an important fermentation product in R1_TS_, the headspace was analyzed periodically with only traces of H_2_ detected and accounting for less than 0.1% of the influent COD. Therefore, H_2_ was deemed not to be a significant fermentation product in R1_TS_. Methane was not detected, confirming that the source of inoculum and the reactor conditions did not favor the enrichment of methanogenic organisms in the bioreactor.

In R2_SR–TS_, approximately 20 g COD L^–1^ of fermentation products were measured during the stable period of bioreactor operation. This represented 38.4 ± 2.0% of effluent COD (Figure 1) and was composed of a mixture of SCFA and MCFA (Figure 2). The SCFA accounted for 8.6 ± 1.0 g COD L^–1^ (16.9 ± 0.2% of effluent COD; *n* = 8) and MCFA corresponded to 8.7 ± 0.9 g COD L^–1^ (17.1 ± 0.1% of effluent COD). In addition to the percent increase of MCFA with respect to R1_TS_, the feeding of SR-TS instead of TS in R2_SR–TS_ while maintaining other operational conditions constant resulted in other observed differences in fermentation products with respect to R1_TS_. For instance, ethanol accumulated in R1_TS_ but not in R2_SR–TS_, and C8 accumulated in the effluent in R2_SR–TS_ but not in R1_TS_.

Reducing the SRT to 1 day in the R3_LowSRT_ bioreactor did not affect the total amount of accumulated fermentation products (ca. 20 g COD L^–1^; 40% of effluent COD) but changed the product profile. While SCFA and MCFA were the main fermentation products in the bioreactors operated with 6-day SRT (R1_TS_ and R2_SR–TS_), the most abundant fermentation product in R3_LowSRT_ was SA, averaging 5.8 ± 2.7 g COD L^–1^ (12.1 ± 0.1% of effluent COD, *n* = 9), which is more than double the concentrations of SA that were observed in the effluent of R1_TS_ and R2_SR–TS_ (Figure 2).

Bioreactor operation at thermophilic conditions, with a slight decrease in pH and maintaining a 6-day SRT, as in the R4_T–pH_ experiment, resulted in a decrease of accumulated fermentation products in the effluent and another switch in the products that accumulated compared with the mesophilic temperature reactors (R1_TS_, R2_SR–TS_, and R3_LowSRT_). The sum of the fermentation products in the R4_T–pH_ accounted for 13.3 ± 0.4 g COD L^–1^ (26.3 ± 0.8% of effluent COD, *n* = 8) and corresponded mostly to C3 and LA. Although C3 production was also observed in other bioreactors, its concentration in R4_T–pH_ was substantially and consistently greater (4.8 ± 0.2 g COD L^–1^) than in the effluent of other bioreactors (Figure 2). LA concentration in TS and SR-TS was ca. 4 g COD L^–1^ (Table 1) and slightly higher in in the thermophilic reactors R4_T–pH_ and R5_T–pH–LowSRT_ (5.3 ± 0.5 g COD L^–1^ and 5.0 ± 1.3 g COD L^–1^, respectively), whereas it was only present at lower concentrations in the mesophilic reactors (R1_TS_, R2_SR–TS_, and R3_LowSRT_). Reducing the SRT to 1 day under thermophilic conditions, as in the R5_T–pH–LowSRT_ experiment, created a condition in which the glycerol present in SR-TS was not completely consumed (Figure 2). We also observed that the sum of fermentation products in R5_T–pH–LowSRT_ was the lowest among all bioreactors, totaling 10.0 ± 1.8 g COD L^–1^ (15.3 ± 1.9% of effluent COD, *n* = 7). Furthermore, compared with the results from R4_T–pH_, the SRT decrease to 1 day in the R5_T–pH–LowSRT_ reactor did not affect the accumulation of LA but eliminated the accumulation of C3.

### Microbial Community Analysis

**FIGURE 3:**
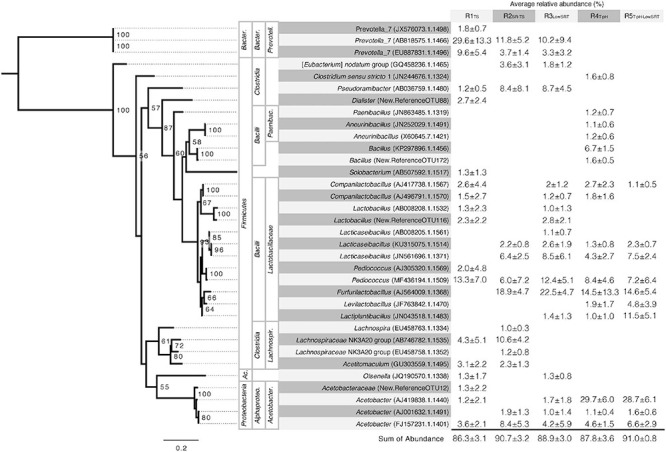
Phylogeny and average relative abundance of operational taxonomic units (OTUs) from the stable period of operation for each reactor. Abundance greater than 1% averaged across five to seven time points is shown. OTU IDs shown in parentheses are assigned as GenBank accession numbers when the alignment of representative sequences to the SILVA reference database was greater than 99%. Sequences below this cut-off are assigned sequentially generated OTU IDs. Taxonomy of OTUs was assigned using the SILVA database when alignment was greater than 99%. Higher taxonomic levels are labeled, left to right, phylum, class, and, family. Abbreviated phyla are as follows: Ac., *Actinobacteria*; Bacter., *Bacteroidetes*. Abbreviated classes are as follows: Alphaproteo., *Alphaproteobacteria*; Bacter., *Bacteroidia*. Abbreviated families are as follows: Acetobacter., *Acetobacteraceae*; Lachnospir., *Lachnospiraceae*; Paenibac., *Paenibacillaceae*; Prevotell., *Prevotellaceae*. Phylogenetic tree is rooted in the genus *Prevotella.* Bootstrap values greater than 50 are shown. Scale bar indicates number of nucleotide substitutions per sequence site.

Genomic DNA was extracted from 88 biomass samples across multiple time points from the five bioreactor experiments. 16S rRNA gene amplicon sequencing by Illumina MiSeq 2 × 300 bp yielded a total of 9.9 Gbp sequence data. Raw reads processed through the QIIME v1.9.1 pipeline were normalized to 30,354 reads per sample. A total of 11,530 unique OTUs were identified across all samples (Supplementary Table 1). Subsequent analyses of the microbial communities focused on the most abundant representative OTUs. A cut-off of an average relative abundance of 1% or greater during the period of reactor stability for each experiment was used, which resulted in 34 highly abundant OTUs accounting for 85% of the total reads in the bioreactor microbial communities (Figure 3). Microbial community data for the inoculum and for each bioreactor experiment are available in Supplementary Tables 2–7, and a summary of relative abundances per sample during the periods of bioreactor stability is presented in Supplementary Figure 2.

The number of OTUs having greater than 1% average relative abundance in each reactor experiment ranged from 10 to 18. Using the same 1% cut-off threshold, the number of abundant OTUs was similar in the inoculum source (i.e., 14 OTUs). The most abundant OTUs in the inoculum (Supplementary Table 2) included members of the genera *Prevotella* and *Cloacibacterium* within the *Bacteroidetes*, members of the *Lactobacillales* and *Clostridiales* order within the *Firmicutes*, and members of the *Arcobacter* genus within *Campylobacterota* and the *Acinetobacter* genus within *Proteobacteria*. However, although representatives of some of these groups were present in the bioreactors, none of the abundant OTUs in the inoculum were representative of the most abundant OTUs in the different reactor communities (Figure 3).

The microbial communities that were enriched in the different bioreactors characteristically had a few (2 or 3) OTUs with relative abundances greater than 10% (Figure 3). *Lactobacillaceae* were ubiquitous in all bioreactors and were represented by four to 10 distinct OTUs accounting for 23–56% of the total reads, respectively (Figure 3). Of these, *Pediococcus* (MF436194.1.1509) was abundant in all bioreactors, and *Furfurilactobacillus* (AJ564009.1.1368), another heterofermentative lactobacillus (Zheng et al., 2020), was the single most abundant lactobacillus OTU at ca. 15–23% relative abundance but only present in the SR-TS-fed reactors. *Clostridia*, including *Pseudoramibacter* (AB036759.1.1480), a putative MCFA-producing bacterium (Holdeman et al., 1967; Scarborough et al., 2020), were prevalent under mesophilic conditions (R1_TS_, R2_SR–TS_, and R3_LowSRT_) at a total relative abundance of 11–27% but were not abundant at thermophilic temperatures. *Prevotella*, a C4-producing *Bacteroidetes* common in the rumen (Esquivel-Elizondo et al., 2017; Fraga et al., 2018), followed a similar trend as the *Clostridia* 14–41% total relative abundance. Thermophilic temperatures (R4_T–pH_ and R5_T–pH–LowSRT_) favored an abundance of *Acetobacter* OTUs with one OTU in particular, AJ419838.1.1440, representing nearly 30% of the reads in either reactor (Figure 3). A more detailed description of the microbial community results is available in the Supplementary Material.

**FIGURE 4:**
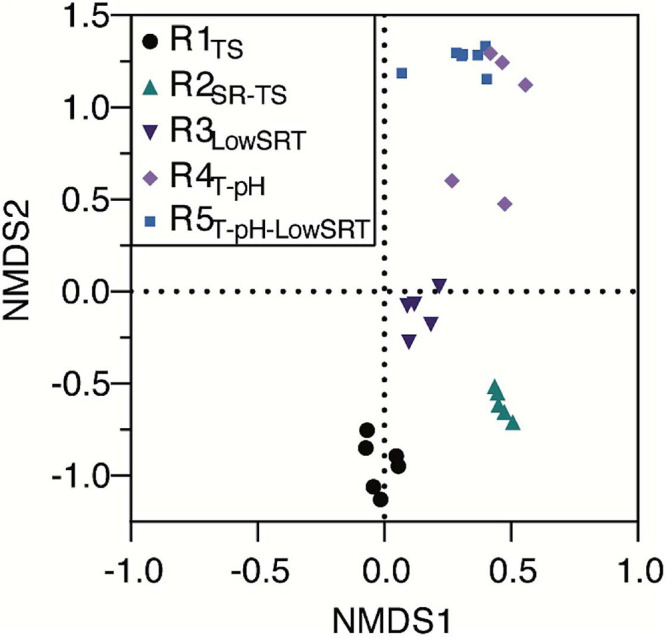
Non-metric multidimensional scaling plot of bioreactor microbial communities during periods of reactor stability when run under different operating conditions.

Although several OTUs were identified as abundant members of the microbial communities in more than one bioreactor (Figure 3), non-metric multidimensional scaling (NMDS) of the microbial community data from all bioreactors (Figure 4) illustrates that each of the five bioreactor experiments was selected for a distinct microbial community. As Figure 4 shows, the data points from the microbial communities from each bioreactor tend to cluster together and not overlap with data points from the other bioreactors, indicating high within-group similarity and low between-group similarity. One exception to this trend is the data from the R4_T–pH_ and R5_T–pH–LowSRT_ experiments, which overlap slightly, indicating a greater similarity between these two communities. A statistical comparison (i.e., ANOSIM) of the microbial communities in the bioreactors reinforces the distinction between experiments (Table 3). The *R*-values of the pairwise comparisons of the bioreactor communities were all close to 1.0, indicating a significantly (*p* < 0.01, for all pairwise comparisons) distinct microbial community under each experimental condition. These data are consistent with the NMDS plot where the one exception to the distinction of the microbial communities from each bioreactor is the pairwise comparison between the R4_T–pH_ and R5_T–pH–LowSRT_ communities where the *R*-value was approximately 0.7. While this still indicates a high level of distinction between the two microbial communities, there is more overlap in these two microbial communities than between the communities from the other bioreactors.

**TABLE 3:** Analysis of similarities (ANOSIM) between microbial communities during the period of greatest stability.

**Reactor experiment sample pair**	***R*^a^**	***p*-Value**	**Number of samples**
R1_TS_–R2_SR–TS_	0.987	0.006	11
R2_SR–TS_–R3_LowSRT_	0.996	0.006	10
R2_SR–TS_–R4_T–pH_	0.996	0.006	10
R3_LowSRT_–R4_T–pH_	0.964	0.009	10
R4_T–pH_–R5_T–pH–LowSRT_	0.744	0.002	12

*Statistics based on 1,000 model permutations.*

*^a^Test statistic, *R*, is on a scale of 0, indistinguishable, to 1, highly distinct.*

## Discussion

Bioreactors, seeded from the same inoculum but operated under different conditions, were enriched with microbial communities that metabolized components of the TS and SR-TS feedstock and accumulated different fermentation products. The five different operating conditions tested resulted in reactor effluents composed of between 13 and 50% different fermentation products including SCFA, MCFA, SA, and LA in varying concentrations. Below, we discuss how this study illustrates the possibilities of value-added products that can be obtained from a renewable and currently underutilized resource by installing a secondary fermentation system in a preexisting starch bioethanol facility.

The possible use of TS as a feedstock for secondary fermentation needs to be balanced with the current use of TS as a component of the DDGS used as animal feed. The presence of oils, fats, and higher-density materials in TS contributes to the nutritional value of DDGS, while the nutritional value from protein is captured in the insoluble WDGs. Although greater quantities of fermentation products were produced with TS than SR-TS, the removal of the higher-density and insoluble materials in TS made the reactors easier to operate on SR-TS while still producing a large amount of fermentation products. Thus, it is possible that the insoluble and more nutritional components of TS could still be marketed for animal feed, which is an important coproduct to the local economies of starch ethanol biorefineries (Lupitskyy et al., 2015), while the more soluble carbohydrate-abundant materials left in SR-TS could be primarily used for gaining value through secondary fermentation. Additionally, the residues after recovery of the secondary fermentation products could still be evaporated and mixed with WDGs to make DDGS (Rosentrater, 2011). Thus, assuming that about 50% of SR-TS is recovered as secondary fermentation products, implementing such process would result in a reduction of only about 25% the amount of TS residues that would reach the evaporation and drying end of the bioethanol facility.

### Importance of Glycerol as a Fermentation Carbon Source

The TS and SR-TS feeds to the bioreactors had a significant concentration of glycerol (Table 1), a feature that likely had an important contribution to shaping the microbial communities in the reactors. The effect of the feed combined with the operational conditions used defined the profiles of fermentation products in the bioreactors. Below, we discuss the effect that glycerol in the feed and the two principal modifications to operational conditions may have had on reactor operation and profiles of fermentation products.

During ethanol fermentation, yeast produces glycerol to maintain redox and osmotic balance. This well-known process is considered a problem in the fuel ethanol industry since it diverts carbon away from the desired end-product (Brumm and Hebeda, 1988; Bideaux et al., 2006). However, in the context of gaining value from the residues of ethanologenic fermentations, glycerol becomes an abundant substrate for the secondary fermentation. Glycerol was quickly consumed under all reactor conditions, except for the R5_T–pH–LowSRT_ experiment (Figure 2). Glycerol metabolism has been demonstrated, or its metabolic potential has been suggested, in microorganisms related to the abundant taxa observed in the bioreactors, including members of the *Clostridia* class and *Lactobacillaceae* family of *Firmicutes* (Veiga da Cunha and Foster, 1992; Alvarez et al., 2004; Cotta and Forster, 2006; Rainey, 2011; Gänzle, 2015; Doi, 2019; Palevich et al., 2019; Veras et al., 2019; Scarborough et al., 2020). In addition to being a coproduct of the fuel ethanol industry, glycerol is a coproduct from the biodiesel industry, which is of growing interest to researchers as a substrate for microbial fermentation of SA and LA (Litsanov et al., 2013; Gao et al., 2016; Murakami et al., 2016; Kuenz et al., 2020; Zheng et al., 2021). The greatest concentration of SA was observed in R3_LowSRT_ at an average of 5.8 ± 2.7 g COD L^–1^ (Figure 2); however, glycerol consumption was similar across all reactor experiments (except R5_T–pH–LowSRT_), and thus, SA production was more likely influenced by another operational condition, rather than influent glycerol concentration. While proteins and amino acids can serve as substrates for microbial SA production (Unden and Kleefeld, 2004; Bevilacqua et al., 2020; Regueira et al., 2020), the concentration of proteins in TS and SR-TS was relatively low (Table 1), and therefore, it is more likely that SA production was more influenced by glycerol than protein concentrations.

Detectable C3 production was observed in all of the bioreactors, except for R5_T–pH–LowSRT_, which had the lowest consumption of glycerol. Thus, we hypothesize C3 production from glycerol metabolism by these bioreactor communities. Propionic acid formation from glycerol is predicted to occur through the decarboxylation of SA from central carbon metabolism (da Silva et al., 2009). Fermentative production of C3 from glycerol as a sole carbon source or a co-substrate is particularly well characterized among the genus *Propionibacterium* in the *Actinobacteria* phylum (Falentin et al., 2010; Zidwick et al., 2013; Gonzalez-Garcia et al., 2017; Ganigué et al., 2019). However, putative relatives of this taxon were not detected in the microbial communities in this study (Supplementary Table 1). Nevertheless, C3 production from glycerol has been observed in other genera and microbial communities (Chen et al., 2016; Gonzalez-Garcia et al., 2017; Ganigué et al., 2019). Production of C3 is potentially important in mixed culture fermentations, such as the experiments presented in this study, because it can serve as a substrate for chain-elongating microorganisms to produce C5 and C7 fatty acids (Grootscholten et al., 2013; de Smit et al., 2019; Candry et al., 2020b). While C6 was consistently the single most concentrated fatty acid produced in the MCFA-producing reactors (R1_TS_, 5.2 ± 3.0 g COD L^–1^; R2_SR–TS_, 5.0 ± 0.9 g COD L^–1^; R3_LowSRT_, 3.0 ± 1.2 g COD L^–1^; Figure 2), C5 and C7 acids also contributed substantially to the total fermentation products in the effluent from these bioreactors. During the period of stable operation, C5 concentration averaged 3.9 ± 2.1 g COD L^–1^, 2.6 ± 0.3 g COD L^–1^, and 2.0 ± 0.7 g COD L^–1^ in R1_TS_, R2_SR–TS_, and R3_LowSRT_, respectively, while C7 was 2.1 ± 1.4 g COD L^–1^, 2.3 ± 0.7 g COD L^–1^, and 1.7 ± 1.3 g COD L^–1^, respectively (Figure 2).

The concentration of odd chain fatty acids observed in this study was higher and was sustained for a longer period of time than has been reported previously in experiments using an acid sludge inoculum from the same wastewater treatment plant, but a more complex feedstock residue from lignocellulosic ethanol biorefining (Scarborough et al., 2018b). One possible explanation for this difference is the glycerol concentration in the feedstock. Glycerol was greater than 20 g COD L^–1^ in TS and SR-TS used in this study and less than 4 g COD L^–1^ in the lignocellulosic residues tested by Scarborough et al. (2018b). Similarly, when comparing the TS used in this study with the TS used in other studies, the glycerol concentration in other TS feedstocks was lower (i.e., less than 10 g COD L^–1^) than observed in the feedstock we used, and the effluent streams from the other studies also contained a lower concentration of C5 or C7 than we observed (Andersen et al., 2015, 2017; Vincent, 2017).

Glycerol is not the only known precursor carbon source for C3 production. Numerous species are able to use LA, or carbohydrates as substrates for C3 fermentation, including *Veillonella*, *Megasphaera*, *Roseburia*, and *Prevotella* (Engels et al., 2016; Louis and Flint, 2016; Gonzalez-Garcia et al., 2017). *Veillonella* and *Megasphaera* are members of the *Veillonellaceae* family within the *Clostridia* class along with *Dialister*, which was an abundant taxon in the R1_TS_ bioreactor (Figure 3). *Roseburia* was present in the inoculum but was not among the abundant taxa in the bioreactors (Supplementary Table 1). *Prevotella* was highly abundant taxa in the mesophilic bioreactors (R1_TS_, R2_SR–TS_, and R3_LowSRT_).

### Reduced Solids Retention Time Operating Conditions Favors Succinic Acid Production

When valorizing underutilized agro-industrial residues and producing commercially and industrially valuable compounds, it is important to achieve high titers of the desired product at a high production rate. Operation of bioreactors using short retention times aims at increasing production rates, which would require smaller tanks and thus contribute to reducing capital costs. In our bioreactor experiments, we observed that at shorter retention time, when the influent was fed to a reactor at a higher flow rate, the feedstocks were metabolized at a faster fermentation rate. We illustrate this with SA production in R3_LowSRT_ when the SRT was reduced stepwise from 6 days to 1 day (Supplementary Figure 3). Prior to SRT reduction (i.e., end of R2_SR–TS_ operation), SA concentration was ca. 2 g COD L^–1^, and the production rate was less than 0.5 g COD L^–1^ day^–1^. With SRT reduction, we observed an increase in SA titer from about 2 g COD L^–1^ to ca. 5–6 g COD L^–1^, which is likely associated with the changes in the microbial community composition. Furthermore, with faster flow rates, the SA production rate increased from less than 0.5 g COD L^–1^ day^–1^ to approximately 5–6 g COD L^–1^ day^–1^ (Supplementary Figure 3).

Historically, the SA market is largely satisfied by production from petroleum. However, more facilities are coming online, which use engineered microorganisms and renewable feedstocks to produce SA (Zeikus et al., 1999; Biddy et al., 2016; Carlson et al., 2016). In some cases, these industrial strains are grown in concentrated solutions of pure carbon sources, such as glucose (Zeikus et al., 1999). However, SA fermentation using industrial and agricultural byproducts such as corn steep solids from wet milling (Rogers et al., 2013) or crude glycerol from biodiesel production has been demonstrated, which is consistent with the potential use of TS or other agro-industrial residues for SA production. Typical examples of industrially relevant bacteria for SA production are *Gammaproteobacteria* including strains of *Actinobacillus*, *Basfia*, *Escherichia*, and *Anaerobiospirillum* (Li et al., 2016, 2018; Nghiem et al., 2017; Zheng et al., 2021), and also *Corynebacterium* from the phylum *Actinobacteria* (Litsanov et al., 2013). While relatives of these genera were absent from the microbial communities in this study (Supplementary Table 1), the abundant taxa from R3_LowSRT_ represent a potential source for cultivating novel strains for SA production (Figure 3).

### Temperature Impact on Thin Stillage Reactor Performance

Results from the analysis of fermentation products in the bioreactor effluent streams and the abundance of microbial community members from the five different experimental conditions suggest that temperature had more of a profound effect on community composition and thereby product formation than did the decrease in SRT (Figure 2 and Supplementary Figure 2). In the case of the bioreactor effluent, the difference when only SRT was changed (e.g., R2_SR–TS_ and R3_LowSRT_) was a decrease in MCFA production and a substantial increase in SA production. However, when temperature was increased, the overall fermentation product concentration decreased by about 25%, MCFA production ceased, and SA production was greatly reduced (Figure 2). Instead, C3 formation increased and LA accumulated in the TS bioreactor incubated at 55°C.

Ammonium hydroxide was selected for pH control to provide an additional benefit as a source of nutritional nitrogen to the microbial communities. Consequently, TAN was in excess in all reactors, although to a lesser extent in the thermophilic reactors (R4_T–pH_ and R5_T–pH–LowSRT_) at 1 g L^–1^ or less, on average (Supplementary Figure 4a). The slightly acidic operating conditions of the bioreactors favor an equilibrium shift toward ammonium ions in solution, resulting in about 1 mg L^–1^ or less FAN (Supplementary Figure 4b). One concern with this strategy was the potential for inhibitory effects that high concentrations of free ammonia could have on the microbial community, particularly since the inhibitory effects of FAN have been found to be exacerbated by increased temperature and pH in anaerobic digestion systems (Jamaludin et al., 2018; Capson-Tojo et al., 2020). While methanogenesis is not an important activity in the fermentations described here, we cannot rule out that FAN concentrations may have had an effect on the type of fermentation products produced at different operational conditions.

Comparing the abundant OTUs between reactors R2_SR–TS_ and R4_T–pH_ provides an approach to evaluate temperature as the main operational variable. This temperature change resulted in the disappearance of the *Prevotella* and the vast majority of the *Clostridiales* as abundant community members and a substantial increase in abundance of *Acetobacter* (Figure 3). Similarly, when comparing the R4_T–pH_ and R5_T–pH–LowSRT_ communities, the only operational change was a reduction in SRT while maintaining thermophilic conditions. While this change in operating conditions resulted in a decrease in *Bacillus* OTU (KP297896.1.1456) abundance and an increase in *Lactiplantibacillus* (JN043518.1.1483) and *Levilactobacillus* (JF763842.1.1470) OTUs, the relative abundance of the dominant OTU (i.e., *Acetobacter*; AJ419838.1.1440) remained constant in the microbial communities.

The effluent from the bioreactor experiments operating at thermophilic temperatures contained the highest observed concentration of LA (Figure 2). Members of the *Lactobacillaceae* are well characterized for the ability to produce LA (Zheng et al., 2020), and several different abundant related OTUs were identified throughout the R4_T–pH_ and R5_T–pH–LowSRT_ microbial communities, including *Companilactobacillus*, *Lacticaseibacillus*, *Pediococcus*, and *Furfurilactobacillus* (Figures 3 and Supplementary Figure 2). However, these OTUs are also present in the microbial communities from the other reactors, so the presence of these OTUs alone does not explain the accumulation of LA. A more likely explanation is that LA accumulated in the higher temperature bioreactors because taxa that otherwise would have consumed LA were absent under these conditions. OTUs identified as *Prevotella*, *Pseudoramibacter*, and *Lachnospiraceae* are present at varying abundances in the communities of the mesophilic bioreactor experiments but absent from the thermophilic communities, and relatives of these taxa are predicted to utilize LA to produce C4 or longer chain acids (Esquivel-Elizondo et al., 2017; Lambrecht et al., 2019; Liu et al., 2020; Scarborough et al., 2020), so their absence could also explain the lack of acids longer than C3 in the effluent of R4_T–pH_ or C2 in the effluent of R5_T–pH–LowSRT_.

One *Acetobacter* OTU (FJ157231.1.1401) was present in the microbiomes from all bioreactors tested in this experiment (ca. 4–8% relative abundance). However, elevated temperatures were selected for an additional highly abundant *Acetobacter* OTU (AJ419838.1.1440, Figure 3). *Acetobacter* is part of the microbial community involved in cacao fermentation, which reaches thermophilic temperatures (Camu et al., 2007; Komagata et al., 2014), and thermotolerant strains have been isolated (Soemphol et al., 2011), so its presence and high abundance in R4_T–pH_ and R5_T–pH–LowSRT_ agrees with prior observations. Although many *Acetobacter* strains oxidize LA (Lisdiyanti et al., 2000; Komagata et al., 2014; Sato et al., 2015), the bioreactors with the greatest abundance of *Acetobacter* (i.e., R4_T–pH_ and R5_T–pH–LowSRT_) accumulated the greatest concentration of LA in the effluent at about 5 g L^–1^. However, it has also been demonstrated that certain *Acetobacter* species when grown on glycerol will produce LA (Kylmä et al., 2004).

## Concluding Remarks

This study provides a comparison of the range of coproducts that can be produced from TS by altering only the bioreactor operating conditions. In this study, we tested the hypotheses that (1) TS could be used as a feedstock to support microbial communities that produce valuable fermentation products and (2) that changes in the microbial community and the product profile could be induced by altering the bioreactor operating conditions. We provided results that support both hypotheses. Two reactors operating at mesophilic temperatures with a 6-day SRT consistently produced a mixture of SCFA and MCFA. Reducing the SRT to 1 day decreased the concentration of fatty acids in favor of increased SA production. OTUs from the *Lachnospiraceae* family were present in the MCFA-producing microbiomes, and a *Pseudoramibacter* OTU was enriched for when SR-TS was fed; however, only the *Pseudoramibacter* persisted under the short SRT conditions. Increasing the temperature eliminated MCFA production altogether, and the reactor effluent contained primarily LA and C3. Decreasing the SRT at elevated temperatures decreased C3 production, and only LA was accumulated. The elevated temperatures were selected against *Pseudoramibacter* and Lachnospiraceae. Relatives of *Acetobacter* and *Bacillus*, which were of low abundance in other reactors, dominated instead. Lactobacillaceae were common in all microbial communities including OTUs related to *Lacticaseibacillus*, *Companilactobacillus*, and *Lactobacillus*. A *Furfurilactobacillus*-related microorganism was abundant in the communities of all SR-TS-fed reactors. One OTU related to *Acetobacter* (FJ157231.1.1401) was relatively abundant under all conditions. These results support our hypotheses and provide a range of potential products that can be obtained industrially from TS by changing reactor operator conditions.

## Data Availability Statement

The datasets presented in this study can be found in online repositories. The names of the repository/repositories and accession number(s) can be found below: https://www.ncbi.nlm.nih.gov/sra/PRJNA719872.

## Author Contributions

NF, TD, and DN designed most of the experiments. All authors contributed ideas to improving the experimental design and contributed to data analysis. NF and NH performed the experiments. NF and DN led the manuscript writing effort. All coauthors contributed to writing and approved the final manuscript.

## Conflict of Interest

The authors declare that the research was conducted in the absence of any commercial or financial relationships that could be construed as a potential conflict of interest.

## Footnotes

1 https://www.biotech.wisc.edu/

2 https://www.arb-silva.de/

3 http://tree.bio.ed.ac.uk/software/figtree/
