# Supplementary material for: Diverse Profile of Fermentation Byproducts From Thin Stillage
Source: Front Bioeng Biotechnol. 2021 Jul 15;9:695306. doi: 10.3389/fbioe.2021.695306 (PMC8320890; doi:10.3389/fbioe.2021.695306)
Supplement: Supplementary file 2 [file Data_Sheet_1.pdf]

Diverse profile of fermentation byproducts from thin stillage

Nathaniel W. Fortney[1], Nathaniel J. Hanson[1,2], Paula R. F. Rosa[1,3], Timothy J. Donohue[1,4], Daniel R. Noguera[1,5*]

[1] Great Lakes Bioenergy Research Center, University of Wisconsin-Madison, Madison, WI, USA

[2] Wisconsin Youth Apprenticeship Program, Department of Workforce Development, Madison, WI, USA

[3] Department of Chemical Engineering, Federal University of São Carlos, São Carlos, SP, Brazil

[4] Department of Bacteriology, University of Wisconsin-Madison, Madison, WI, USA

[5] Department of Civil and Environmental Engineering, University of Wisconsin-Madison, Madison, WI, USA

*Corresponding author: Daniel R. Noguera, noguera@engr.wisc.edu, 608-263-7783.

SUPPLEMENTAL RESULTS

Detailed community analysis

In the $R1_{TS}$ experiment, two highly abundant *Prevotella* 7-related OTUs, (AB818575.1.1466 and EU887831.1.1496) accounted for 27.7±13.4% and 9.6±5.4% of the 16S rRNA gene sequence reads per sample, respectively. *Prevotella* are common rumen inhabitants and have been shown to produce C4 from LA and carbohydrates (Esquivel-

Elizondo et al., 2017; Fraga et al., 2018). *Pediococcus* (MF436194.1.1509) was the most prominent *Lactobacillaceae* in the $R1_{TS}$ bioreactor at 13.3±7.0% relative abundance, whereas the abundance of other genera of this family was much lower. Like other heterofermentative *Lactobacillaceae,* pediococci produce LA and C2 from carbohydrates (Dicks & Endo, 2014; Zheng et al., 2020). Two *Lachnospiraceae* OTUs, *Acetitomaculum* (GU303559.1.1495) and *Lachnospiraceae* NK3A20 group (AB746782.1.1535), were present at approximately 3-4% relative abundance. *Acetitomaculum* are another typical rumen inhabitant noted for C2 production from carbohydrates (Greening & Leedle, 1989; Li et al., 2012; Rainey, 2011). Unique to the abundant OTUs in $R1_{TS}$ was an OTU related to *Dialister* (New.ReferenceOTU88), a genus of Clostridia which is noted for the ability to metabolize succinate and produce C3 (Morotomi et al., 2008; Wade, 2011), present at 2.7±2.4% relative abundance. Conversely, an *Acetobacter*-related OTU (FJ157231.1.1401), present at 3.6±2.1% in the $R1_{TS}$ community, was also present as an abundant microbial community member in all five bioreactors (Fig. 3). *Acetobacter* are common in fermented foods, but have also been observed in other mixed culture fermentations (Andersen et al., 2017). They are typically noted for the ability to produce C2 from carbohydrates and other carbon sources including glycerol, and subsequent mineralization of C2 (Cleenwerck et al., 2008; Cleenwerck et al., 2002; Komagata et al., 2014).

Shifts in the microbial community occurred when the solids were removed from TS in the $R2_{SR\text{-}TS}$ experiment, including the selection of an OTU related to *Furfurilactobacillus* (AJ564009.1.1368), a heterolactic fermenter, at 18.9±4.7% relative abundance (Fig. 3). A second lactobacillus that was enriched to a lower abundance (6.4±2.5% relative abundance) in the $R2_{SR\text{-}TS}$ reactor was related to *Lacticaseibacillus* (JN561696.1.1371), a

homolactic fermenter. Both lactobacilli have been isolated from diverse substrates such as cereal grain fermentations and corn liquor (Zheng et al., 2020). The *Lachnospiraceae* NK3A20 group (AB746782.1.1535) OTU that was present in $R1_{TS}$ was also abundant in $R2_{SR-TS}$, at 10.6±4.2% abundance. Additional *Clostridia* including OTUs related to *Pseudoramibacter* (AB036759.1.1480), which are known or predicted to produce MCFA from carbohydrates (Holdeman et al., 1967), glycerol and LA (Scarborough et al., 2020), and related to the [*Eubacterium*] *nodatum* group (GQ458236.1.1465) were abundant under the $R2_{SR-TS}$ reactor conditions at 8.4±8.1% and 3.1±3.1%, respectively. Two *Prevotella* 7 OTUs (EU887831.1.1496 and AB818575.1.1466), initially observed in $R1_{TS}$, decreased in relative abundance, to 3.7±1.5% and 11.8±5.2%, respectively.

Decreasing the SRT to 1 day in $R3_{LowSRT}$ resulted in the washout of the *Lachnospiraceae*-related OTUs, but other OTUs from the *Clostridia* remained abundant in the microbial community, such as *Pseudoramibacter* (AB036759.1.1480) and [*Eubacterium*] *nodatum* group (GQ458236.1.1465) at 8.7±4.5% and 1.8±1.3% relative abundance, respectively (Fig. 3). Additionally, *Pediococcus* (MF436194.1.1509) remained a prominent OTU in the microbial community at 12.4±5.1% relative abundance. *Companilactobacillus* species are a frequent presence in food fermenting communities (Zheng et al., 2020), and the two related OTUs (AJ496791.1.1570 and AJ417738.1.1567) from $R3_{LowSRT}$ maintained a consistent low-level abundance at 1.2±0.7% and 2.0±1.2%, respectively. Conversely, *Furfurilactobacillus* (AJ564009.1.1368) was the most abundant lactobacillus in the community, at 22.5±4.7%. The *Prevotella* 7 OTUs further decreased in abundance to ca. 10% (AB818575.1.1466) and 3% (EU887831.1.1496).

Operating the bioreactor at thermophilic temperatures (55 °C) had a pronounced effect on the composition of the microbial community in $R4_{T\text{-}pH}$. *Prevotella* OTUs were absent from the abundant members of the microbial community and *Clostridiales* OTUs were reduced in relative abundance as compared to the reactors operating at mesophilic temperatures (Fig. 3). Instead, *Acetobacter* OTUs accounted for approximately 35% of the reads in $R4_{T\text{-}pH}$, and a single OTU (AJ419838.1.1440) dominated at 29.7±6.0% relative abundance. The second most abundant OTU corresponded to *Furfurilactobacillus*, while other lactobacilli were present at lower abundances. In addition, a *Bacillus* OTU (KP297896.1.1456) was enriched in $R4_{T\text{-}pH}$, with 6.8±1.5% relative abundance.

The $R5_{T\text{-}pH\text{-}LowSRT}$ microbial community had only 11, the fewest number of, OTUs greater than 1% relative abundance among all the bioreactors. In general, the prominent OTUs in the $R5_{T\text{-}pH\text{-}LowSRT}$ bioreactor were similar to those observed in $R4_{T\text{-}pH}$, corresponding to a microbial community dominated by *Acetobacter* (AJ419838.1.1440, 28.7±6.1%) with a few lesser abundant *Lactobacillaceae*-related OTUs (Fig. 3). Two additional *Lactobacillaceae* OTUs, *Lactiplantibacillus* (JN043518.1.1483) at 11.5±5.1% relative abundance, and *Levilactobacillus* (JF763842.1.1470), a lactobacillus associated with beer-spoilage (Zheng et al., 2020), was present at 4.8±3.9%.

SUPPLEMENTAL FIGURES

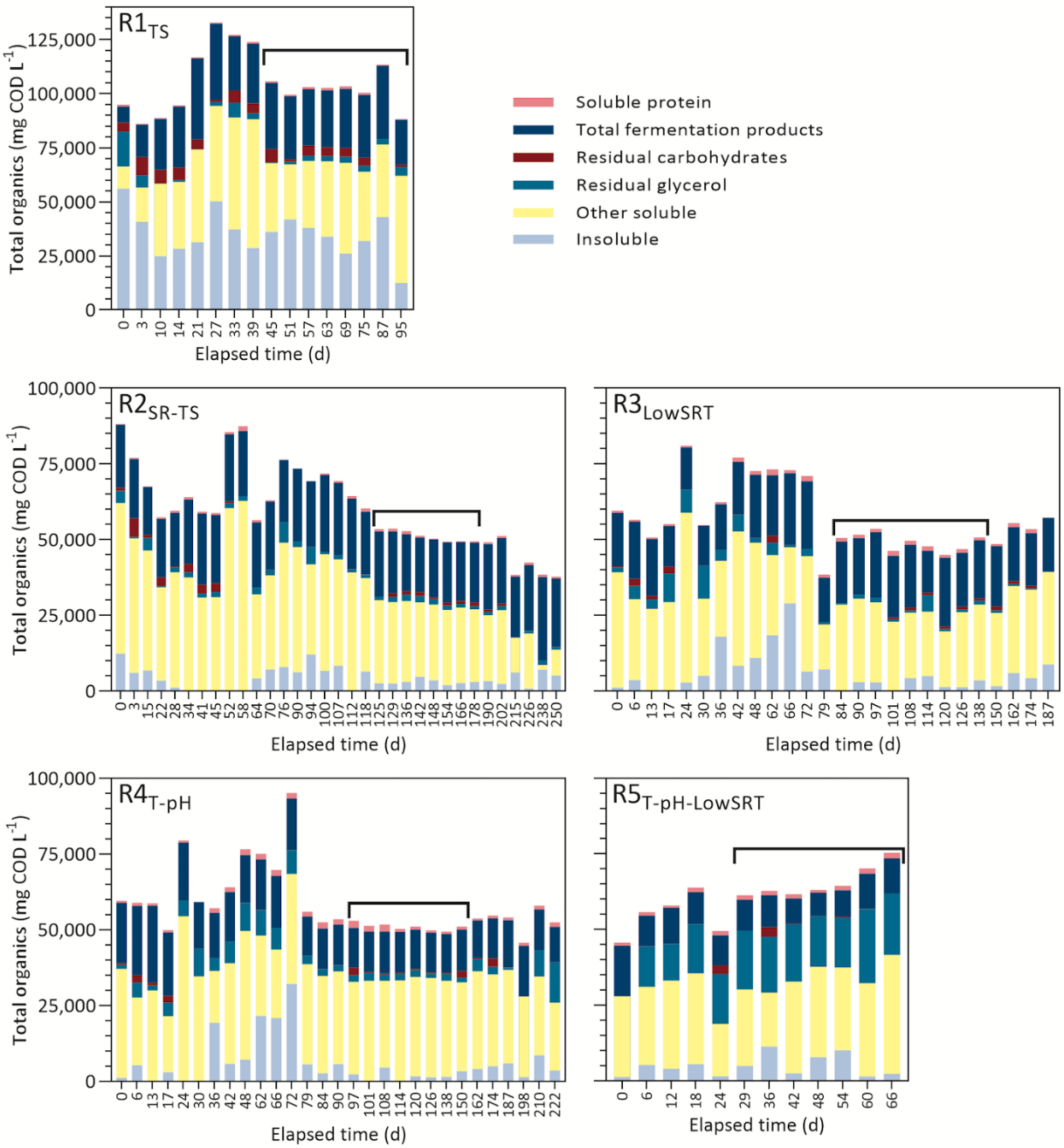

Figure S1. Dynamics of bioreactors ran under different operating conditions. $R3_{LowSRT}$ operated at a 1 d SRT starting at 48 d. $R4_{T\text{-}pH}$ was operated at 55°C starting at 24 d, pH was reduced to 4.5 by 54 d, and increased to 5.0 by 120 d. $R5_{T\text{-}pH\text{-}LowSRT}$ was initially at thermophilic temperatures and operated at a 1 d SRT at 19 d. Insoluble portion of the total organics represents the difference between total and soluble COD of the effluent and can be

inferred to be accumulated microbial biomass, with the exception of $R1_{TS}$ which also contains influent-derived solids. Other soluble COD represents the remaining measured COD that was uncharacterized by other analytical methods plus metabolites detected at trace concentrations. Total carbohydrates are the sum of unconsumed glucose, xylose, and cellobiose remaining in the effluent. Total fermentation products represent the sum of all carboxylic acids and ethanol. Brackets mark a window of approximately 50 d during which bioreactor operation was more stable following the initial dynamic phase.

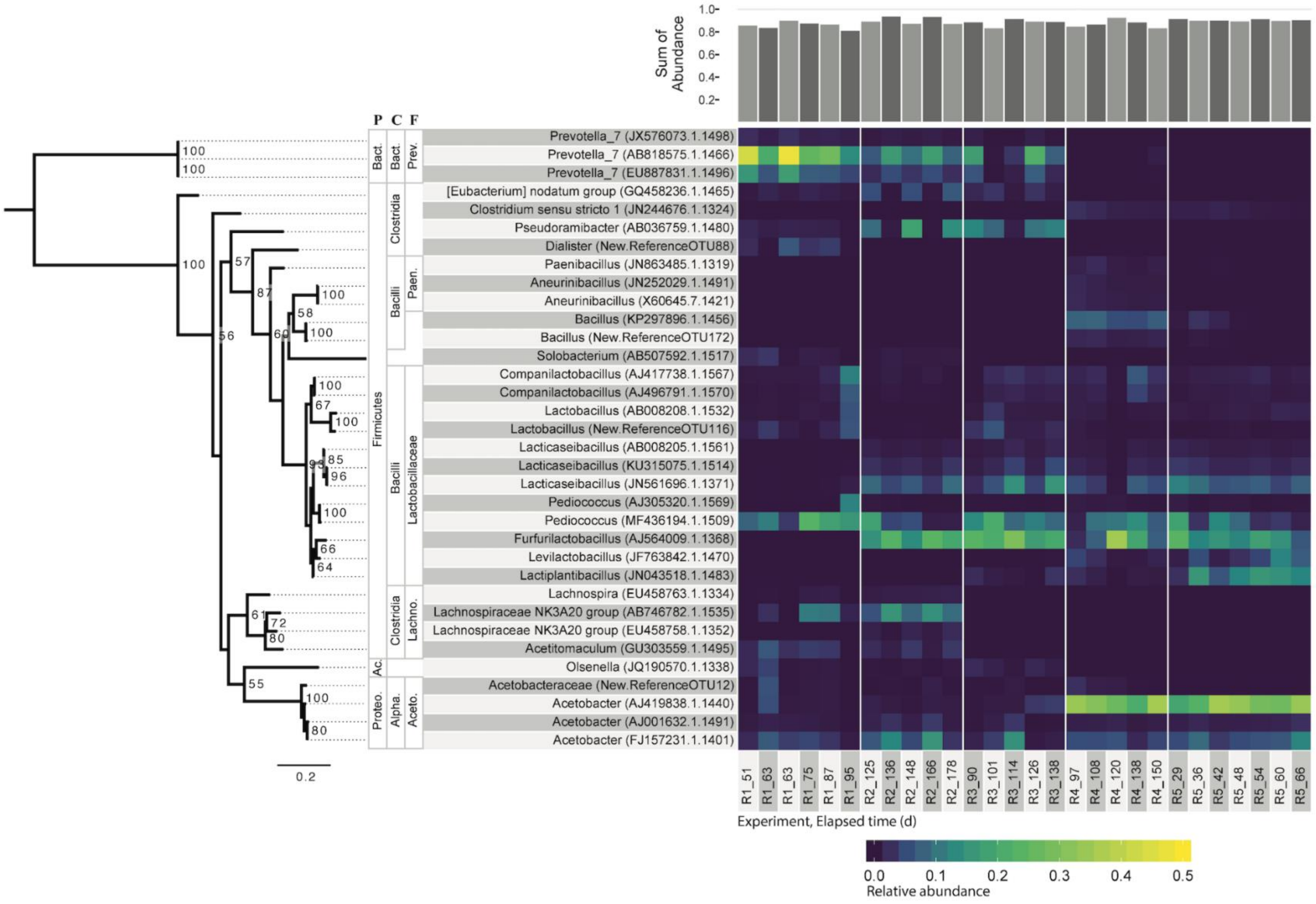

Sum of Abundance
P C F
Bact.
Firmicutes
Ac.
Proteo.
Clostridia
Bacilli
Alpha.
Prev.
Paen.
Lactobacillaceae
Lachno.
Aceto.
Prevotella_7 (JX576073.1.1498)
Prevotella_7 (AB818575.1.1466)
Prevotella_7 (EU887831.1.1496)
[Eubacterium] nodatum group (GQ458236.1.1465)
Clostridium sensu stricto 1 (JN244676.1.1324)
Pseudoramibacter (AB036759.1.1480)
Dialister (New.ReferenceOTU88)
Paenibacillus (JN863485.1.1319)
Aneurinibacillus (JN252029.1.1491)
Aneurinibacillus (X60645.7.1421)
Bacillus (KP297896.1.1456)
Bacillus (New.ReferenceOTU172)
Solobacterium (AB507592.1.1517)
Companilactobacillus (AJ417738.1.1567)
Companilactobacillus (AJ496791.1.1570)
Lactobacillus (AB008208.1.1532)
Lactobacillus (New.ReferenceOTU116)
Lacticaseibacillus (AB008205.1.1561)
Lacticaseibacillus (KU315075.1.1514)
Lacticaseibacillus (JN561696.1.1371)
Pediococcus (AJ305320.1.1569)
Pediococcus (MF436194.1.1509)
Furfurilactobacillus (AJ564009.1.1368)
Levilactobacillus (JF763842.1.1470)
Lactiplantibacillus (JN043518.1.1483)
Lachnospira (EU458763.1.1334)
Lachnospiraceae NK3A20 group (AB746782.1.1535)
Lachnospiraceae NK3A20 group (EU458758.1.1352)
Acetitomaculum (GU303559.1.1495)
Olsenella (JQ190570.1.1338)
Acetobacteraceae (New.ReferenceOTU12)
Acetobacter (AJ419838.1.1440)
Acetobacter (AJ001632.1.1491)
Acetobacter (FJ157231.1.1401)
R1_51 R1_63 R1_63 R1_75 R1_87 R1_95 R2_125 R2_136 R2_148 R2_166 R2_178 R3_90 R3_101 R3_114 R3_126 R3_138 R4_97 R4_108 R4_120 R4_138 R4_150 R5_29 R5_36 R5_42 R5_48 R5_54 R5_60 R5_66
Experiment, Elapsed time (d)
Relative abundance
0.2

Figure S2. OTUs with greater than 1% average relative abundance in the microbial community during periods of stable reactor conditions (ca. 50 d) for five different bioreactor operational conditions: R1, $R1_{TS}$; R2, $R2_{SR\text{-}TS}$; R3, $R3_{LowSRT}$; R4, $R4_{T\text{-}pH}$; R5, $R5_{T\text{-}pH\text{-}LowSRT}$. Taxonomy was assigned by aligning representative sequences to the SILVA Database (version 138.1). OTU IDs, in parenthesis, are represented by accession numbers where alignment to the database was greater than 99% or were otherwise assigned *de novo*. Phylogenetic tree is rooted in the OTUs representing the genus *Prevotella*. Bootstrap values greater than 50 are shown. Scalebar indicates the number of nucleotide substitutions per sequence site. Phyla abbreviations: Bact., *Bacteroidetes*; Ac., *Actinobacteria*; Proteo., *Proteobacteria*. Class (C) abbreviations: Bact., *Bacteroidia*; Alpha., *Alphaproteobacteria*. Family (F) abbreviations: Prev., *Prevotellaceae*; Paen., *Paenibacillaceae*; Lachno., *Lachnospiraceae*; Aceto., *Acetobacteriaceae.*

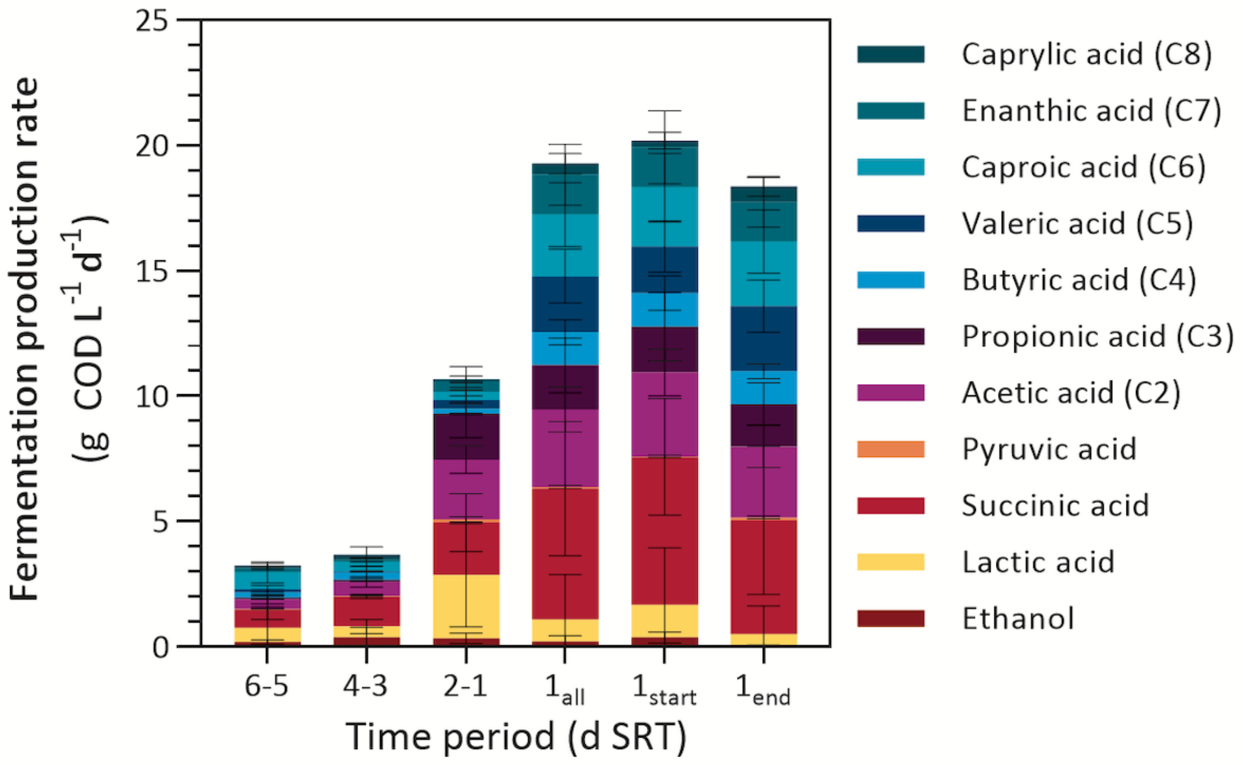

Figure S3. Fermentation product formation rate in $R3_{LowSRT}$ from a 6 d SRT to a 1 d SRT, averaged across three samples per time period. The production rate during the 1 d SRT phase was averaged across all samples at this retention time (n=18), the first half of the 1 d SRT phase (n=9), and last half of 1 d SRT operation (n=9).

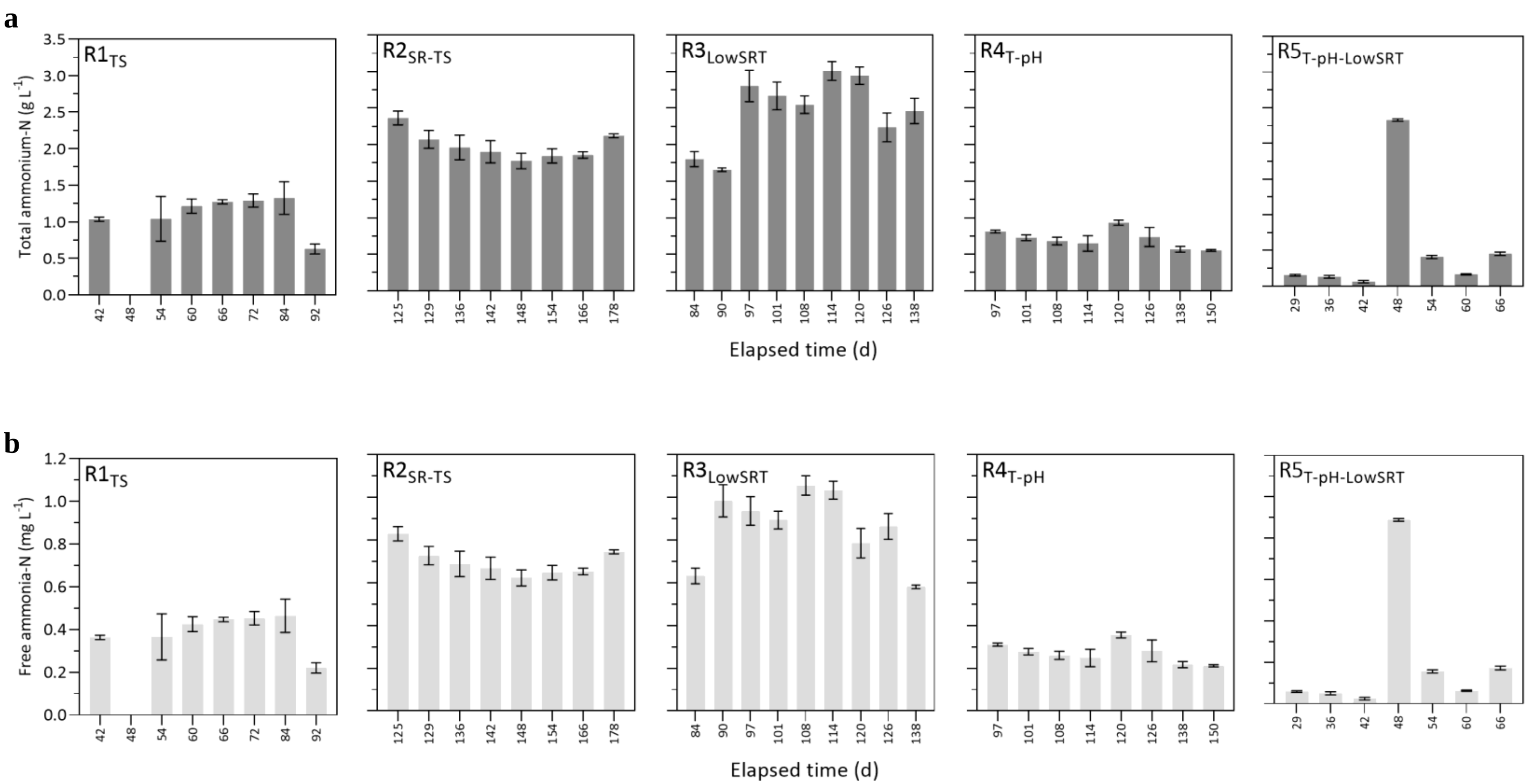

Figure S4. Total ammonium nitrogen (TAN) concentration (a; sum of NH_4^+-N and NH_3-N), and free ammonia nitrogen (FAN) concentration (b; ideal equilibrium calculation) during indicated periods of reactor stability. Note the difference in units between the two figures.

SUPPLEMENTAL REFERENCES

Andersen, S.J., De Groof, V., Khor, W.C., Roume, H., Props, R., Coma, M., Rabaey, K. 2017. A Clostridium Group IV Species Dominates and Suppresses a Mixed Culture Fermentation by Tolerance to Medium Chain Fatty Acids Products. *Frontiers in Bioengineering and Biotechnology*, **5**, 8189-10.

Cleenwerck, I., Gonzalez, Á., Camu, N., Engelbeen, K., De Vos, P., De Vuyst, L. 2008. *Acetobacter fabarum* sp. nov., an acetic acid bacterium from a Ghanaian cocoa bean heap fermentation. *International Journal of Systematic and Evolutionary Microbiology*, **58**(Pt 9), 2180-2185.

Cleenwerck, I., Vandemeule, K., Janssens, D., Swings, J. 2002. Re-examination of the genus *Acetobacter*, with descriptions of *Acetobacter cerevisiae* sp. nov. and *Acetobacter malorum* sp. nov. *International Journal of Systematic and Evolutionary Microbiology*, **52**(5), 1551-1558.

Dicks, L., Endo, A. 2014. The Family *Lactobacillaceae*: Genera Other than *Lactobacillus*. in: *The Prokaryotes*, (Eds.) E. Rosenberg, E.F. DeLong, S. Lory, E. Stackebrandt, F. Thompson, Springer-Verlag. Berlin Heidelberg, pp. 203-212.

Esquivel-Elizondo, S., Ilhan, Z.E., Garcia-Peña, E.I., Krajmalnik-Brown, R. 2017. Insights into Butyrate Production in a Controlled Fermentation System via Gene Predictions. *mSystems*, **2**(4), 201-13.

Fraga, M., Fernández, S., Perelmuter, K., Pomiés, N., Cajarville, C., Zunino, P. 2018. The use of *Prevotella bryantii* 3C5 for modulation of the ruminal environment in an ovine model. *Brazilian Journal of Microbiology*, **49**(Supplement 1), 101-106.

Greening, R.C., Leedle, J.A.Z. 1989. Enrichment and isolation of *Acetitomaculum ruminis*, gen. nov., sp. nov.: acetogenic bacteria from the bovine rumen. *Archives of Microbiology*, **151**, 399-406.

Holdeman, L.V., Cato, E.P., Moore, W.E.C. 1967. Amended Description of *Ramibacterium alactolyticum* Prévot and Taffanel with Proposal of a Neotype Strain. *International Journal of Systematic Bacteriology*, **17**(4), 323-341.

Komagata, K., Iino, T., Yamada, Y. 2014. The Family *Acetobacteraceae*. 4 ed. in: *The Prokaryotes - Alphaproteobacteria and Betaproteobacteria*, (Eds.) E. Rosenberg, E.F. DeLong, S. Lory, E. Stackebrandt, F. Thompson, Vol. 2, Springer-Verlag. Berlin Heidelburg, pp. 3-78.

Li, M., Zhou, M., Adamowicz, E., Basarab, J.A., Guan, L.L. 2012. Characterization of bovine ruminal epithelial bacterial communities using 16S rRNA sequencing, PCR-DGGE, and qRT-PCR analysis. *Veterinary Microbiology*, **155**(1), 72-80.

Morotomi, M., Nagai, F., Sakon, H., Tanaka, R. 2008. Dialister succinatiphilus sp. nov. and Barnesiella intestinihominis sp. nov., isolated from human faeces. *International Journal of Systematic and Evolutionary Microbiology*, **58**(Pt 12), 2716-2720.

Rainey, F.A. 2011. Family V. Lachnospiraceae fam. nov. in: *Bergey's Manual of Systematic Bacteriology*, (Eds.) P. De Vos, G.M. Garrity, D. Jones, N.R. Krieg, W. Ludwig, F.A. Rainey, K.-H. Schleifer, W.B. Whitman, Vol. 3, Springer Science & Business Media. New York, NY, pp. 921-968.

Scarborough, M.J., Myers, K.S., Donohue, T.J., Noguera, D.R. 2020. Medium-Chain Fatty Acid Synthesis by "*Candidatus* Weimeria bifida" gen. nov., sp. nov., and "*Candidatus* Pseudoramibacter fermentans" sp. nov. *Applied and Environmental Microbiology*, **86**(3), 2796.

Wade, W.G. 2011. Genus XII. Dialister. 2 ed. in: *Bergey's Manual of Systematic Bacteriology , Vol. 3: The Firmicutes*, (Eds.) P. Vos, G. Garrity, D. Jones, W. Ludwig, F.A. Rainey, K.-H. Schleifer, W.B. Whitman, Vol. 3, Springer. New York, pp. 1080-1082.
Zheng, J., Wittouck, S., Salvetti, E., Franz, C.M.A.P., Harris, H.M.B., Mattarelli, P., O'Toole, P.W., Pot, B., Vandamme, P., Walter, J., Watanabe, K., Wuyts, S., Felis, G.E., G√§nzle, M.G., Lebeer, S. 2020. A taxonomic note on the genus Lactobacillus: Description of 23 novel genera, emended description of the genus Lactobacillus beijerinck 1901, and union of Lactobacillaceae and Leuconostocaceae. *International Journal of Systematic and Evolutionary Microbiology*, **70**(4), 2782-2858.
